# Unnatural amino acids increase activity and specificity of synthetic substrates for human and malarial cathepsin C

**DOI:** 10.1007/s00726-013-1654-2

**Published:** 2014-01-01

**Authors:** Marcin Poreba, Marko Mihelic, Priscilla Krai, Jelena Rajkovic, Artur Krezel, Malgorzata Pawelczak, Michael Klemba, Dusan Turk, Boris Turk, Rafal Latajka, Marcin Drag

**Affiliations:** 1 Division of Bioorganic Chemistry, Faculty of Chemistry, Wroclaw University of Technology, Wybrzeze Wyspianskiego 27, 50-370 Wrocław, Poland; 2 Department of Biochemistry and Molecular and Structural Biology, Jozef Stefan Institute, Ljubljana, Slovenia; 3 Department of Biochemistry, Virginia Tech, Blacksburg, VA 24061 USA; 4 Laboratory of Chemical Biology, Faculty of Biotechnology, University of Wrocław, ul. Joliot-Curie 14a, 50-383 Wrocław, Poland; 5 Faculty of Chemistry, University of Opole, ul. Oleska 48, 45-052 Opole, Poland

**Keywords:** Cysteine protease, Non-proteinogenic, Unnatural amino acid, Substrate library, Fluorogenic substrate

## Abstract

Mammalian cathepsin C is primarily responsible for the removal of N-terminal dipeptides and activation of several serine proteases in inflammatory or immune cells, while its malarial parasite ortholog dipeptidyl aminopeptidase 1 plays a crucial role in catabolizing the hemoglobin of its host erythrocyte. In this report, we describe the systematic substrate specificity analysis of three cathepsin C orthologs from *Homo sapiens* (human), *Bos taurus* (bovine) and *Plasmodium falciparum* (malaria parasite). Here, we present a new approach with a tailored fluorogenic substrate library designed and synthesized to probe the S1 and S2 pocket preferences of these enzymes with both natural and a broad range of unnatural amino acids. Our approach identified very efficiently hydrolyzed substrates containing unnatural amino acids, which resulted in the design of significantly better substrates than those previously known. Additionally, in this study significant differences in terms of the structures of optimal substrates for human and malarial orthologs are important from the therapeutic point of view. These data can be also used for the design of specific inhibitors or activity-based probes.

**Electronic supplementary material:**

The online version of this article (doi:10.1007/s00726-013-1654-2) contains supplementary material, which is available to authorized users.

## Introduction

The specificity ratio between enzyme orthologs or homologs is one of the most important factors in terms of drug design or specific enzyme activity monitoring using chemical probes. For example, there are several families of proteases, such as caspases, cathepsins or aminopeptidases, which are able to cleave the same peptide sequences, which significantly complicate their targeting with specific chemical tools, such as substrates or activity-based probes. This goal is even more complicated when trying to design specific molecules for monitoring enzyme orthologs, for example, in the case of parasite infection in humans. An excellent example of this difficulty is human cathepsin C and its malarial ortholog dipeptidyl aminopeptidase 1 (DPAP1).

Cathepsin C (DPPI, EC 3.4.14.1, dipeptidyl peptidase I) is a lysosomal cysteine protease expressed in the majority of mammalian tissues (Tallan et al. 1952). It is considered a major coordinator for the activation of several serine proteases (cathepsin G, cytotoxic lymphocyte derived granzymes A and B, neutrophil elastase) in immune/inflammatory cells (Turk et al. 2001; Pham and Ley 1999; McGuire et al. 1993). Defects in cathepsin C expression lead to several disorders, including Haim–Munk and Papillon–Lefevre syndromes (Hart et al. 1999, 2000). Other studies report the involvement of cathepsin C in cytotoxic lymphocyte-mediated apoptosis, angiogenesis or host immune defense (Gocheva and Joyce 2007; Adkison et al. 2002). Structurally, human cathepsin C is a homotetramer (~200 kDa) comprising four identical catalytically active subunits (Dahl et al. 2001; Turk et al. 2001). Each subunit contains a light chain, a heavy chain and an exclusion domain. Mechanistically, cathepsin C is a classic exopeptidase, which trims dipeptides from the N-terminus of peptide substrates.

The malarial ortholog of mammalian cathepsin C, dipeptidyl aminopeptidase 1 (DPAP1), is a cysteine protease, which most efficiently hydrolyzes amide bonds at acidic pH (Wang et al. 2011). DPAP1 is located in food vacuoles and plays the role of an intermediate protease between the endopeptidase and aminopeptidase activities (Kudo et al. 2012). Specifically, according to current hypotheses, DPAP1 hydrolyzes the peptide sequences generated by three classes of endopeptidases: aspartic proteases (plasmepsins), cysteine proteases (falcipains) and metalloproteases (falcilysin). This leads to short peptide fragments, which are further hydrolyzed into single amino acids in the vacuole by the metal-dependent M1-family aminopeptidase, *Pf*A-M1 (Ragheb et al. 2011). These amino acids are either used by parasites for protein synthesis or are released by the parasite into the surrounding media (Krugliak et al. 2002). In contrast to humans, parasite genomes contain another two DPAP1-related enzymes, DPAP2 and DPAP3. DPAP3 inhibition leads to the blockage of parasite egress (Arastu-Kapur et al. 2008). A recent work by Tanaka et al. demonstrates that DPAP2 is active in gametocyte. It also showed that the DPAP2 KO in *P. falciparum* or *P. berghei* has no effect on parasite development, thus indicating that DPAP2 is not essential (Tanaka et al. 2013).

The implication of cathepsin C and DPAP1 in pathological disorders makes both enzymes very interesting medicinal targets. To date, human cathepsin C has especially been investigated, with several chemical approaches leading to potent substrates, inhibitors and activity-based probes (Yuan et al. 2006; Guay et al. 2010). DPAP1 has been less extensively investigated. The substrate specificity of both enzymes, interrogated with a combinatorial library of fluorogenic dipeptides containing natural amino acids, revealed some differences in the recognition of the S1 and S2 subsites, but few significant differences between the enzymes have been found (Wang et al. 2011).

In this report, we have designed and synthesized a fluorogenic dipeptide substrate library containing all natural amino acids (except cysteine, which is prone to oxidation) and several structurally different unnatural amino acids. We hypothesized that the application of such a broad range of different amino acid structures would help to identify more significant differences in the optimal substrates recognized by human and malarial cathepsin C and to design more active substrates in terms of kinetic parameters. To obtain better insight into cathepsin C orthologs, in addition to human and malarial cathepsin C, we also analyzed the substrate specificity of the bovine (*Bos Taurus*) ortholog.

The approach presented here can be generally used for the substrate specificity screening of other diaminopeptidases. When the structure of an enzyme is not available, information from the library can be used to predict the size of and preferences for the S1 and S2 pockets and can be further applied to the design of specific substrates or inhibitors. We also demonstrate that this approach allows the differentiation of enzyme orthologs from different species, yielding information about evolutionary changes.

## Materials and methods

### General

Fmoc-Rink-amide AM polystyrene resin (mesh 100–200, 0.64 mmol/g), piperidine, *O*-(benzotriazol-1-yl)-*N*,*N*,*N*′,*N*′-tetramethyluronium hexafluorophosphate (HBTU) and trifluoroacetic acid (TFA) were purchased from Iris Biotech GmbH. Anhydrous *N*,*N*-dimethylformamide (DMF) was purchased from J. T. Baker. Dichloromethane (DCM), methanol (MeOH) and diethyl ether (Et_2_O) were purchased from POCH S.A. (Poland). Fmoc-protected amino acids were purchased from Sigma Aldrich, Iris Biotech GmbH, Fluka and Novabiochem. *O*-(7-azabenzotriazol-1-yl)-N,N,N′N′-tetramethyluronium hexafluorophosphonate (HATU) was purchased from Novabiochem. 2,4,6-trimethylpyridine (collidine), *N*,*N*-diisopropylethylamine (DIPEA), triisopropylsilane (TIPS), DEAE-Sepharose, Sephadex G-200, 2-mercaptoethanol, diisopropyl phosphorofluoridate (DFP), bovine serum albumin and EDTA-Na_2_ were purchased from Sigma Aldrich (USA). Molecular weight calibration markers for gel filtration and protein markers for SDS-PAGE were purchased from Bio-Rad (USA). Gly-Phe-pNA was a gift from Dr. Maciej Makowski, Department of Chemistry, University of Opole (Opole, Poland). All chemicals and solvents were used without further purification. 7-Fmoc-aminocoumarin-4-acetic acid was synthesized in our laboratory according to the procedure described previously (Maly et al. 2002).

### Human, bovine and malarial cathepsin C expression and purification

Human cathepsin C was expressed according to the procedure described elsewhere (Dahl et al. 2001). Cathepsin C was activated by cathepsin L (molar ratio 20:1, respectively) in the following buffer: 20 mM citric acid, 150 mM NaCl, 1 mM EDTA and 5 mM DTT, pH 4.5. Cathepsin C was activated for 4 h. The protein mixture was loaded onto a preparative HiLoad Superdex 200 size-exclusion column using fast protein liquid chromatography (AKTA Purifier, 1.6 cm × 60 cm, GE Healthcare, Sweden) equilibrated with 50 mM sodium acetate, 1 mM EDTA and 300 mM NaCl, pH 5.5, at a flow rate of 1.2 mL/min and 5 °C. The protein was analyzed by SDS-PAGE, and fractions containing cathepsin C were collected and concentrated to 5 μM. Active site titration was carried out as described in Online Resource 2. The enzyme was 65 % active. Cathepsin C concentrations and *k*_cat_ values are given per enzyme complex.

Bovine cathepsin C (DPP I; EC 3.4.14.1) was purified from bovine spleen after acid extraction, heat treatment, ammonium sulfate fractionation, gel filtration chromatography and ion-exchange chromatography. Purification was carried out according to the method developed by McDonald et al. and supplemented with an ion-exchange chromatography step on a column of DEAE-Sepharose (Ken McDonald et al. 1972). The purified enzyme showed a native molecular mass of ~200 kDa by Sephadex G-200 column chromatography. Enzyme concentration was calculated based on total protein.

Purified recombinant *P. falciparum* dipeptidyl aminopeptidase 1 (rDPAP1) was generated as described in Wang et al. (2011). Enzyme concentration was calculated based on total protein. DPAP1 concentrations, and k_cat_ values are given per enzyme complex.

### Synthesis of the substrate library

NH2-ACC resin was prepared by the reaction of Amide-Rink resin with the 7-Fmoc-aminocoumarin-4-acetic acid. The resin was first swollen in anhydrous dichloromethane for an hour and washed with DMF, and the Fmoc-protecting group was removed by 20 % piperidine/80 % DMF (25, 5, 5 min). This prepared resin was washed three times with DMF. Next, deprotected resin was dissolved in DMF, and Fmoc-ACC-OH (2.0 eq.), HBTU (2.0 eq.) and DIPEA (2.0 eq.) were added. The mixture was agitated for 24 h, filtered and washed (3 times with DMF). The resin was then redissolved in DMF, and the second coupling was performed with Fmoc-ACC-OH (1.0 eq.), HBTU (1.0 eq.) and DIPEA (1.0 eq.). The mixture was agitated for the next 24 h, filtered and washed (3 times with DMF). The substitution level after the second coupling was >98 %. The Fmoc-ACC resin was deprotected with 20 % piperidine/80 % DMF (25, 5, 5 min), filtered, washed (3 times with DMF, 3 times with DCM and 3 times with MeOH) and dried over P_2_O_5_. The NH2-ACC resin was used to construct both the P1 and the P2 fluorogenic substrate libraries.

#### P1 library

Dried NH_2_-ACC resin was split into 36 portions (100 mg each) and placed into the wells of a 96-well semiautomatic FlexChem synthesizer. The resin was then swollen in anhydrous DCM for an hour. Next, the NH_2_-ACC resin was filtered, washed (3 times with DMF) and solvated in DMF. An individual Fmoc-amino acid-OH (2.5 eq.), HATU (2.5 eq.) and collidine (2.5 eq.) were sequentially added to the wells, and the reaction was agitated for 24 h, filtered and washed (3 times with DMF). The second coupling was as follows: individual Fmoc-amino acid-OH (1.0 eq.), HATU (1.0 eq.) and collidine (1.0 eq.). The mixture was shaken for the next 24 h, filtered and washed (3 times with DMF). The Fmoc-protecting group was removed by 20 % piperidine/80 % DMF (25, 5, 5 min), and the resin was washed three times with DMF. In the P2 position, l-methionine was fixed. The resin was swollen in anhydrous DMF, and Fmoc-l-Met-OH (2.5 eq.), HBTU (2.5 eq.) and DIPEA (2.5 eq.) were added. The mixture was shaken for 3 h, filtered and washed (3 times with DMF). Next, the Fmoc-protecting group was removed as previously described. The resin was filtered, washed three times with DMF, three times with DCM, three times with MeOH and dried over P_2_O_5._ Finally, the dry NH_2_-l-Met-X-ACC resin was obtained. The cold mixture of TFA (95 %), water (2.5 %) and TIPS (2.5 %) was used to remove the P1 library from the resin (agitating for 2 h). Each single substrate was precipitated in cold ether and centrifuged. After decantation, each substrate was dissolved in DMSO and purified by HPLC on a Waters M600 solvent delivery module with a Waters M2489 detector system using a semi-preparative Waters Spherisorb S10ODS2 column. The solvent composition was as follows: phase A (water/0.1 % TFA) and phase B (acetonitrile/water 80 %/20 % (v/v) with 0.1 % of TFA). The purity of each substrate was confirmed by analytical HPLC using a Waters Spherisorb S5ODS2 column. All compounds were at least 95 % pure. Each of the 36 dipeptidyl fluorogenic substrates was lyophilized, weighed and dissolved in DMSO to a final concentration of 20 mM. Finally, the molecular weight of each substrate was confirmed by ESI-MS analysis.

#### P2 library

Dry NH_2_-ACC resin was split into 57 portions (100 mg each) and placed into the wells of a 96-well semiautomatic FlexChem synthesizer. Then, the resin was swollen in anhydrous DCM for an hour. Next, the NH_2_-ACC resin was filtered, washed (3 times with DMF) and solvated with DMF. The P1 position was fixed with l-homophenylalanine as follows: Fmoc-l-hPhe-OH (2.5 eq.), HATU (2.5 eq.) and collidine (2.5 eq.) were added to each well, and the mixtures were shaken for 24 h, filtered, washed (3 times with DMF) and redissolved in DMF. In the second coupling, Fmoc-l-hPhe-OH (1.0 eq.), HATU (1.0 eq.) and collidine (1.0 eq.) were added to each well, and the mixtures were agitated for the next 24 h, filtered and washed as previously described. The substitution level after the second coupling was >95 % (HPLC analysis). Next, Fmoc-l-hPhe-ACC resin was deprotected with 20 % piperidine/80 % DMF (25, 5, 5 min), and the resin was washed three times with DMF. The P2 position was substituted with individual Fmoc-amino acid-OH as follows: Fmoc-amino acid-OH (2.5 eq.), HBTU (2.5 eq.) and DIPEA (2.5 eq.) were added to individual wells containing solvated (DMF) NH_2_-l-hPhe-ACC resin. The mixtures were agitated for 3 h, filtered and washed (3 times with DMF). The Fmoc-protecting group was removed with 20 % piperidine/80 % DMF (25, 5, 5 min), and the resin was washed three times with DMF, three times with DCM and three times with MeOH and dried over P_2_O_5_. The cold mixture of TFA (95 %), water (2.5 %) and TIPS (2.5 %) was used to remove the P2 library from the resin. Each single substrate was precipitated in cold ether and centrifuged. After the decantation and lyophilization, each substrate was dissolved in DMSO to a final concentration of 20 mM. There was no need to further purify the substrates because the substitution level after coupling l-hPhe was at least 95 %, and the P2 coupling reaction occurs with a 100 % yield. The purity of each single substrate was confirmed by analytical HPLC using a Waters Spherisorb S5ODS2 column. The solvent composition was as follows: phase A (water/0.1 % TFA) and phase B (acetonitrile/water 80 %/20 % (v/v) with 0.1 % of TFA).

### Assay of the substrate library

The P1 and P2 fluorogenic substrate libraries were screened against cathepsin C from two mammals (*Homo sapiens* and *Bos taurus*) and one protozoan parasite (*Plasmodium falciparum*). The P1 library (NH_2_-l-Met-**X**-ACC) consists of 36 individual compounds, and the P2 library (NH_2_-**X**-l-hPhe-ACC) consists of 57 individual compounds. Human cathepsin C was assayed in the following buffer: 100 mM sodium acetate, 100 mM NaCl, 1 mM EDTA, and 5 mM DTT, pH 5.5. Bovine spleen cathepsin C was activated in 170 mM NaCl solution containing 1 mM EDTA and 1 mM DTT at 37 °C for 0.5 h. The enzyme was then assayed in 0.1 M acetate buffer containing 30 mM NaCl, 1 mM EDTA and 1 mM DTT, pH 5.0. Buffers for the screening of mammalian cathepsin C orthologs were prepared at 23 °C, and assays were conducted at 37 °C.

Assays of DPAP1 were conducted in 50 mM Na-MES, pH 6, 30 mM NaCl, 1 mM EDTA, 2 mM DTT and 0.1 % Triton X-100 at 25 °C. Each library compound was assayed at a concentration of 1 μM. The library was screened using a DPAP1 concentration of 2 nM. To obtain reliable rates with the most efficiently cleaved substrates, the enzyme concentration needed to be reduced to 0.5 nM. Rates from these reactions were multiplied by a factor of four to enable comparison with rates obtained with 2 nM enzyme. The background rate was determined in an enzyme blank containing 1.0 μM Val-Arg-ACC (Wang et al. 2011) and was subtracted from all enzymatic rates.

Before being added to the substrate, cathepsins were preincubated at 37 °C for 30 min. The final library concentration was 1 μM. Enzyme concentrations were between 1 and 5 nM.

The hydrolysis of ACC substrates was monitored continuously with an excitation wavelength of 355 nm and an emission wavelength of 460 nm using a Spectra MAX Gemini EM fluorimeter (Molecular Devices). The total time of each assay was between 5 and 15 min. From each single experiment, the linear portion of the progress curve was used to calculate the final substrate rate of hydrolysis and reported as the relative fluorescence unit per second (RFU/s). Each experiment was repeated at least three times, and the standard error of measurements was calculated. The average value for each substrate was compared with the best-cleaved substrate of a given library. All data were presented on a two-dimensional graph, where the *x*-axis represents individual fluorogenic substrates and the *y*-axis represents the production of relative fluorescence units set to 100 % for Met-Arg-ACC in P1 and Arg-hPhe in P2 library.

### Determination of kinetic parameters (*k*_cat_, *K*_m_, and *k*_cat_/*K*_m_) for individual substrates

Selected substrates were analyzed against human cathepsin C with the above assay buffers. Before being added to the substrate, all enzymes were preincubated at 37 °C for 30 min. The ACC final concentration was calculated by a total digestion assay for human cathepsin C. In each measurement, ten independent substrates with known concentrations were chosen, and the average value was calculated. To measure the *K*_m_ value, eight different concentrations of the given substrates and constant enzyme concentrations were used. The reaction volume was 100 μL, and the enzyme concentration was 1.0 nM for human cathepsin C. All experimental conditions were as above. The hydrolysis of ACC substrates was monitored as in the previous section. The total time of each assay was between 10 and 30 min. All experiments were repeated at least three times, and the average value with standard deviation was calculated. The concentration of DMSO in each experiment was <1 % (v/v). For DPAP1, kinetic analyses were conducted in triplicate as previously described using 2 nM DPAP1 (Wang et al. 2011). We have also calculated the kinetic parameters for human cathepsin C using optimal cathepsin C (NH_2_-Abu-Nle(OBzl)-ACC) and DPAP1 (Pip-Lys-ACC) substrates at different pH, DTT and NaCl concentrations as well as in optimal condition for each enzyme buffer. They are attached in Online Resource.

## Results

### Design of the P1 and P2 dipeptide libraries

**Fig. 1:**
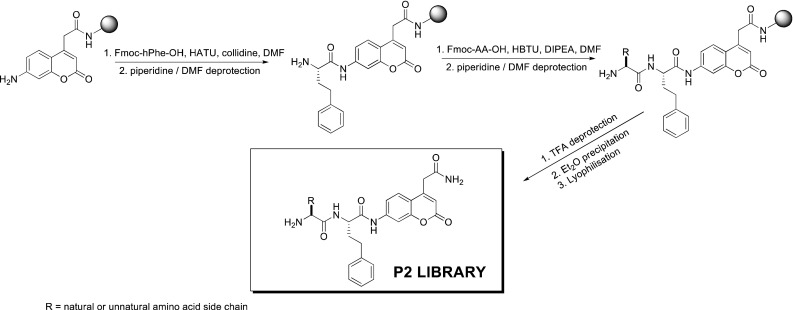
Synthesis and structure of P2 library

To use unnatural amino acids in our approach, we have not used the classic positional scanning substrate combinatorial library (PS-SCL) methodology due to its use of only natural amino acids. In our approach, we have synthesized the P2 library of substrates by fixing in the P_1_ position l-hPhe (l-homo-phenylalanine), an amino acid described in previous reports as one of those most preferred by cathepsin C (Li et al. 2009). l-hPhe was coupled (double coupling) with more than 95 % yield to ACC fluorophore (7-amino-4-carbamoylmethylcoumarin) linked to Rink-amide resin, according to previously described methodology (Fig. 1) (Maly et al. 2002).

We used the ACC fluorophore as the leaving group because of its convenience in solid phase synthesis (Maly et al. 2002). Next, NH_2_-l-hPhe-ACC was split, and the parallel coupling of Fmoc-protected amino acids using a semiautomatic FlexChem synthesizer was performed, yielding individual substrates after cleavage.

In this library, which consisted of 57 individual substrates, we used all natural amino acids (except l-cysteine due to its susceptibility to oxidation), several l-amino acid enantiomers (d-amino acids) and a broad range of unnatural amino acids, of which the structures were chosen to cover a spectrum of the possible interactions in the S2 pocket of human cathepsin C (full structures are in Online Resource 1). The purity of the substrates was confirmed using analytical HPLC. Finally, we screened the whole library at a substrate concentration of 1 μM, which was sufficiently below the lowest *K*_m_ of all tested substrates to ensure that velocity data are proportional to *k*_cat_/*K*_m_.

**Fig. 2:**
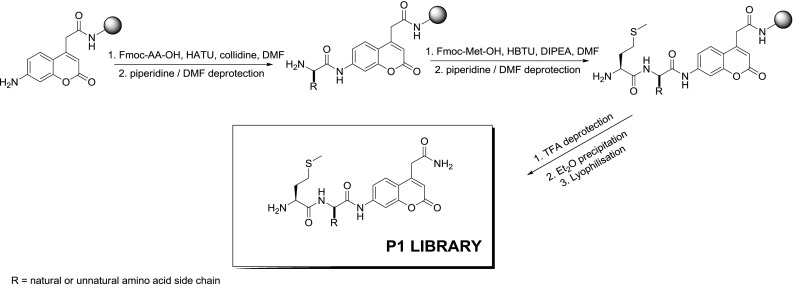
Synthesis and structure of the P1 library

Having determined the P2 preference of cathepsin C, we next designed a library to screen the substrate specificity in the S1 pocket of this enzyme. In the first step, we obtained the ACC fluorophore linked to Rink-amide resin. Next, this resin was split, and double coupling of the first amino acid was performed using previously described methodology with a semiautomatic FlexChem synthesizer. In the P2 position, we fixed the optimal natural amino acid determined in the previous step, l-Met (Fig. 2). To build this library consisting of 36 individual substrates, we applied all natural amino acids (except l-cysteine) and several unnatural amino acids. However, based on previous reports, we applied mostly unnatural amino acids with bulky and hydrophobic side chains [Bpa, Bip, Nle(*O*-Bzl), hPhe, Glu(Bzl)] (full structures are in Online Resource 1) (Li et al. 2009). Finally, all the substrates were cleaved from the resin and purified using preparative HPLC and analyzed using analytical HPLC.

Exactly as described above, we determined the preliminary conditions (*K*_m_) of all cleaved substrates and performed the parallel screening of the library at a final substrate concentration of 1.0 μM.

### Substrate specificity analysis of human and bovine cathepsin C and DPAP1

**Fig. 3:**
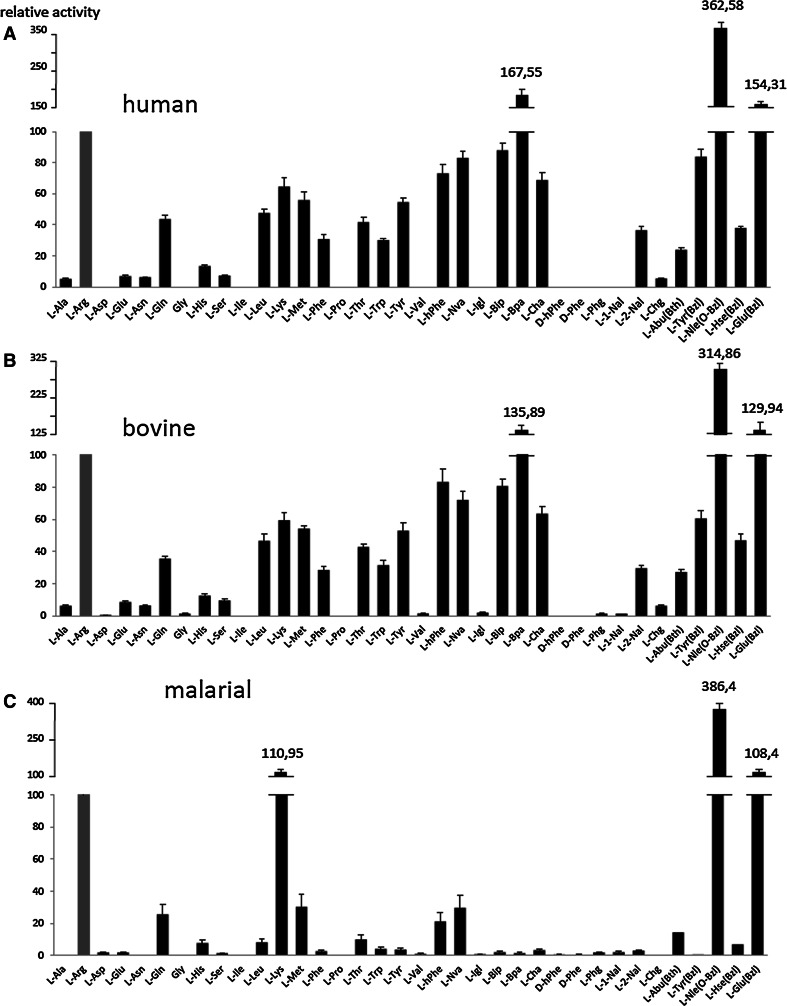
Substrate specificity of human and bovine cathepsin C and malarial DPAP1 in the S1 pocket (substrate concentration 1 μM, human and bovine cathepsin C—3 nM, rDPAP1—2 nM). Proteinogenic and unnatural amino acid abbreviations are shown on the *x*-axis. The *y*-axis represents the average relative activity as a percentage of the L-Arg substrate activity. All the results were normalized to L-Arg. All structures and information about fluorogenic substrates are in Online Resource 1

Bovine cathepsin C is often used to mimic human cathepsin C. We applied our library to compare both enzymes directly in terms of substrate specificity. The analysis of the S1 pocket preferences of mammalian cathepsin C demonstrates that these enzymes recognize exactly the same residues at almost the same level (Fig. 3a, b). The most preferred amino acids can be assigned to one of the following groups: hydrophobic [Nle(*O*-Bzl), Bpa, Bip, Tyr(Bzl), Glu(Bzl), hPhe], basic (Arg, Lys) or aliphatic (Nva, Met, Leu). This finding demonstrates that the S1 pocket size is much larger than the natural amino acids and can very easily accommodate more bulky residues, clearly confirming a conserved level of structure organization of these enzymes.

This observation is also in agreement with data published by Li et al. (2009), which demonstrated that some unnatural amino acids (l-hPhe or l-Bpa) bind much better than l-Phe (Fig. 3). Among natural amino acids, the ones best tolerated by human and bovine cathepsin C were Arg, Lys, Gln and Met. These data are in quite good agreement with previously published data by Wang et al. (2011), who applied a combinatorial library approach. However, it needs to be underlined that the best natural amino acid (Arg) was recognized only at ~20 % compared to the best unnatural derivative from our library (l-Nle(*O*-Bzl)).

Malarial DPAP1 substrate specificity in the S1 pocket is much more restricted compared to the mammalian orthologs tested here. DPAP1 preferentially recognizes and hydrolyzes such amino acids like Nle(*O*-Bzl), Lys, Glu(Bzl), Arg, Met, Gln, Thr, hPhe and Nva (Fig. 3c). These data are in quite good agreement with the substrate specificity of mammalian orthologs. The most striking difference can be observed in the case of large and bulky unnatural amino acids (Bip, Bpa, Cha). DPAP1 very minimally hydrolyzes these derivatives, which clearly demonstrates the difference in S1 pocket preferences between mammalian and parasite orthologs.

**Fig. 4:**
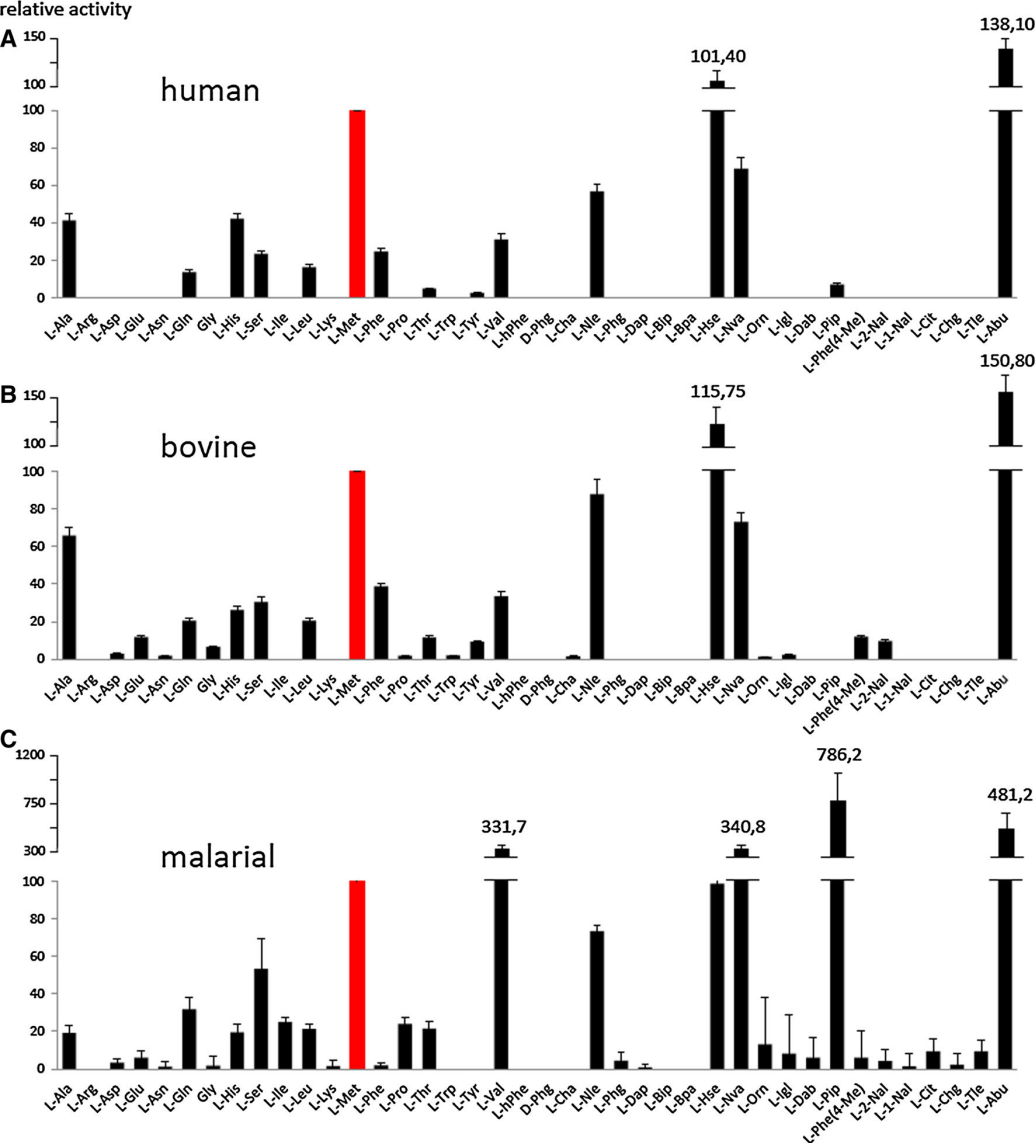
Substrate specificity of human and bovine cathepsin C and malarial DPAP1 in the S2 pocket (substrate concentration 1 μM, human and bovine cathepsin C—3 nM, DPAP1—2 nM). Proteinogenic and unnatural amino acid abbreviations are shown on the *x*-axis. The *y*-axis represents the average relative activity as a percentage of the L-Met substrate activity. d-amino acids, which were not recognized by any of the tested enzymes, are not shown here. All the results were normalized to methionine. All structures and information about fluorogenic substrates are in Online Resource 1

The analysis of the S2 pocket of both mammalian orthologs demonstrates, similar to the case of the S1 pocket, a very high level of agreement in the activity and tolerance of amino acids (Fig. 4a, b).

The amino acids best recognized by both enzymes were Abu, Hse, Met, Nva, Nle and Ala. All these amino acids are rather small and have aliphatic chains. Bulky and hydrophobic side chains were practically unrecognized by either enzyme in the S2 pocket. No activity of human cathepsin C toward amino acids with basic side chains (Arg, Lys, Orn, Dap and Dab) was observed, in agreement with previously published data (Wang et al. 2011). Additionally, none of the d-amino acids was recognized and hydrolyzed by mammalian orthologs, which indicates the high stereospecificity of both enzymes around the S2 binding pocket (structures in Online Resource 1). Although we found slightly different overall ratios from the report by Wang et al. (2011) with regard to natural amino acids, our values are in quite good agreement in terms of overall amino acid preferences. The differences observed can result from various screening conditions or the composition of the library.

The substrate specificity analysis of malarial DPAP1 demonstrates a very striking difference from mammalian orthologs. L-Pip, a six-membered cyclic unnatural homolog of proline with one extra methylene residue, was the best tolerated in the S2 pocket. Substrates with this residue were recognized at least twice as well by DPAP1 than the second best-recognized amino acid, Abu, and almost two-and-a-half times better than the best-recognized natural amino acid, Val. Data obtained for natural amino acids are also in quite good agreement with these previously published (Wang et al. 2011). Another interesting finding was that this amino acid was barely recognized by either mammalian ortholog, which gives hope in the design of specific substrates or inhibitors. A great example here are the data obtained with the inhibitor specificity profile reported by Arastu-Kapur et al., where a library of dipeptide vinyl-sulfone inhibitors containing natural and non-natural amino acids in the P2 position was screened against DPAP1, DPAP3 and the falcipains. In this study, inhibitor with Pro in P2 position was found to be specific for DPAP1 (Arastu-Kapur et al. 2008). In addition to L-Pip, other natural and unnatural amino acids were recognized at a decent rate by DPAP1. These amino acids were Abu, Val, Met, Nva, Hse and Ala. Similarly, as in the case of mammalian orthologs, d-amino acids were not recognized and hydrolyzed by DPAP1.

### Detailed kinetic analysis of fluorogenic substrates of human cathepsin C and DPAP1

In the first step of the analysis, we have focused on the kinetic parameters (*K*_m_, *k*_cat_, *k*_cat_/*K*_m_) of human cathepsin C for several substrates selected from the P1 and P2 libraries. In the P1 library, we found that the highest enzyme efficiency (*k*_cat_/*K*_m_) for human cathepsin C (5.3 × 10^6^ s^−1^ M^−1^) was observed with Met-Nle(*O*-Bzl)-ACC, which is in good agreement with the library screening data. Very good kinetic values were also observed for the other dipeptide substrates with bulky and hydrophobic unnatural amino acids in the P1 position, such as Met-Glu(Bzl), Met-Bip and Met-Bpa (Table 1).

**Table 1:** Kinetic parameters (*K*_m_, *k*_cat_, *k*_cat_/*K*_m_) of selected substrates for human cathepsin C from the P1 library (NH_2_-l-Met-**X**-ACC). Each measurement was repeated at least three times

NH_2_-Met-**X**-ACC		Human cathepsin C		
X: code/name	X: structure	*K*_m_ μM	*k*_cat _s^−1^	*k*_cat_/*K*_m_ × 10^5^ s^−1^ M^−1^
Arg (arginine)		7.49 ± 0.61	12.80 ± 0.08	17.2 ± 0.15
hPhe (homophenylalanine)		3.46 ± 0.09	5.63 ± 0.27	16.1 ± 0.15
Bip (biphenylalanine)		4.11 ± 0.13	6.28 ± 0.61	14.7 ± 0.33
Bpa (4-benzoyl-phenylalanine)		5.43 ± 0.25	15.56 ± 0.87	28.3 ± 0.58
Nle(*O*-Bzl) (6-benzyloxynorleucine)		1.68 ± 0.11	9.27 ± 0.35	53.2 ± 2.55
Glu(Bzl) (glutamic acid benzyl ester)		2.18 ± 0.05	6.05 ± 0.26	27.8 ± 0.18

Analysis of the kinetic parameters for human cathepsin C in the P2 position demonstrates a high preference for small aliphatic side chains. The highest enzyme efficiency (*k*_cat_/*K*_m_), 1.8 × 10^6^ s^−1^ M^−1^ (human) was found with an unnatural amino acid derivative, Abu (Table 2). Other preferred derivatives in the S2 pocket were Hse and Met. All these values are in very good agreement with the library screening data (Fig. 4).

**Table 2:** Kinetic parameters (*K*_m_, *k*_cat_, *k*_cat_/*K*_m_) of selected substrates for human cathepsin C from the P2 library (NH_2_-**X**-l-hPhe-ACC)

NH_2_-**X**-hPhe-ACC		Human cathepsin C		
X: code/name	X: structure	*K*_m_ μM	*k*_cat_ s^−1^	*k*_cat_/*K*_m_ × 10^5^ s^−1^ M^−1^
Ala (alanine)		24.8 ± 1.78	13.3 ± 0.88	5.44 ± 0.29
Leu (leucine)		19.6 ± 1.46	5.00 ± 0.11	2.44 ± 0.04
Met (methionine)		3.6 ± 0.12	5.59 ± 0.14	15.2 ± 0.75
Nle (norleucine)		9.9 ± 0.78	8.35 ± 0.31	8.16 ± 0.16
Hse (homoserine)		8.8 ± 0.66	11.4 ± 0.45	12.5 ± 0.12
Abu (homoalanine)		6.3 ± 0.58	10.4 ± 0.68	17.7 ± 0.82

Each experiment was repeated at least three times

To validate the substrate preferences in the P1 and P2 pockets of human cathepsin C, we designed and synthesized fluorogenic dipeptide substrates that contain the best-recognized amino acids, Nle(*O*-Bzl) in the P1 position and Abu in the P2 position. In parallel, we synthesized and routinely used a commercial substrate for mammalian cathepsin C, which has phenylalanine in the P1 position and glycine in the P2 position. Next, we directly compared all of the kinetic parameters of both substrates and found that the substrate we designed with unnatural amino acids is more than 400 times better than Gly-Phe in terms of *k*_cat_/*K*_m_ values. A further analysis of the kinetic parameters demonstrates that the difference between both substrates is primarily seen in the *K*_m_ value, which is significantly higher for Gly-Phe (Table 3). Interestingly, the turnover number (*k*_cat_) did not differ as greatly between these substrates (approximately, five times greater for our substrate than for Gly-Phe).

**Table 3:** Kinetic parameters (*K*_m_, *k*_cat_, *k*_cat_/*K*_m_) of the best substrate identified and a commercial substrate for human cathepsin C. Each experiment was repeated at least three times

ACC substrate	NH_2_-Abu-Nle(*O*-Bzl)-ACC	NH_2_-Gly-Phe-ACC
Structure		
*K*_m_ μM	1.88 ± 0.11	167.2 ± 5.3
*k*_cat_ s^−1^	17.8 ± 0.56	3.66 ± 0.11
*k*_cat_/*K*_m_ × 10^5^ s^−1^ M^−1^	94.5 ± 0.34	0.22 ± 0.013

**Fig. 5:**
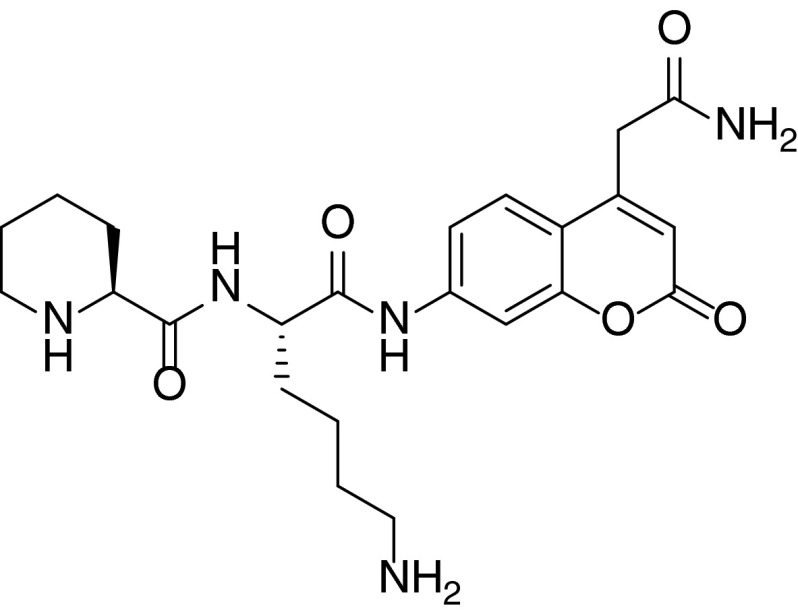
Structure of the optimal substrate for malarial DPAP1

Finally, we compared the kinetic parameters between human and parasite orthologs with the hope of finding significant differences that would allow us to differentiate the enzymes. Our primary aim was to find a sequence that would be very efficiently recognized by DPAP1, but significantly less so by the human ortholog. Sequence alignment analysis reveals that parasitic DPAP1 shares 24 and 26 % identity with human and bovine orthologs, respectively. This was reflected in significant differences in the library screening data, where the malarial parasite ortholog had substrate specificity distinct from mammalian orthologs. The most striking difference was observed in the P2 position, where the unnatural Pip derivative was recognized very efficiently by DPAP1, but minimally by mammalian orthologs. The comparison of kinetic parameters for the P2 library sequence Pip-hPhe confirms the library-based data. DPAP1 hydrolyzed this sequence three times more efficiently (*k*_cat_/*K*_m_—3.22 × 10^5^ s^−1 ^M^−1^) than the human ortholog (*k*_cat_/*K*_m_—1.16 × 10^5^ s^−1^ M^−1^) (Table 4). To further improve the efficiency of the substrates, we designed a DPAP1 substrate, Pip-Lys, with an optimal sequence in the P2 positions and Lys in P1 position (Fig. 5). Since Nle(*O*-Bzl) is the most preferred by all three tested proteases in P1, in search for specific DPAP1 sequence we decided to use Lys, which is not predominantly preferred by human orthologs and is second best for DPAP1. Kinetic analysis reveals that this substrate is approximately two times better in terms of its *k*_cat_/*K*_m_ value (6.59 × 10^5^ s^−1^ M^−1^) than the sequence with hPhe in the P1 position (3.22 × 10^5^ s^−1^ M^−1^) (Table 4). Pip-Lys-ACC is also more specific toward DPAP1. It is more than six times more active (*k*_cat_/*K*_m_) than human cathepsin C (Table 4).

**Table 4:** Kinetic parameters (*K*_m_, *k*_cat_, *k*_cat_/*K*_m_) of selected substrates for human and malarial cathepsin C

Substrate	*K*_m_, μM		*k*_cat_, s^−1^		*k*_cat_/*K*_m_ × 10^−5^, s^−1^ M^−1^	
	Human	Malarial	Human	Malarial	Human	Malarial
Pip-hPhe-ACC	90.9 ± 4.91	0.67 ± 0.14	10.8 ± 0.16	0.22 ± 0.004	1.16 ± 0.11	3.22 ± 0.53
Pip-Lys-ACC	77.4 ± 5.35	0.18 ± 0.01	7.45 ± 0.41	0.12 ± 0.0004	0.96 ± 0.07	6.59 ± 0.37

## Discussion

Due to the implication of human cathepsin C in Papillon–Lefevre disease and Haim-Munk syndrome or inflammatory diseases, and the involvement of its malarial ortholog DPAP1 in the hemoglobin digestion pathway, both enzymes are considered interesting medical targets (Deu et al. 2010; Guay et al. 2010; Klemba et al. 2004). It is especially intriguing that during parasite infection, both enzymes are found in the human (DPAP1 is found in human red blood cells infected with *P. falciparum* parasite, while human cathepsin C is a lysosomal protease), and therefore molecules designed to specifically reach the DPAP1 active site must significantly differentiate between human and parasite orthologs. Due to the involvement of human cathepsin C in activation of several serine proteases in inflammatory or immune cells, cross-reactivity of potential inhibitor of DPAP1 with human ortholog might result in detrimental side effects. Studies published to date regarding the differentiation between human cathepsin C and DPAP1 have only been partially successful; new tools are required to solve this problem (Wang et al. 2011).

In our studies, we have applied a new approach for the selection of very active and selective substrates for these aminodipeptidases. First, we have designed and synthesized a library of individual fluorogenic dipeptide substrates to probe the S1 and S2 pockets of the enzymes. The major advantage of this library is its composition, which includes both natural and unnatural amino acids. Unnatural amino acids were selected from different structural groups of compounds (aliphatic, aromatic, bulky hydrophobic, d-amino acids, cyclic) to cover all possible interactions in the binding pockets. This library allowed us to directly compare three orthologs of cathepsin C, human, bovine and malarial parasite. We found that the human and bovine orthologs have almost identical substrate specificity in their S1 and S2 pockets. They prefer bulky, aromatic residues in the P1 position and rather small and aliphatic amino acids in the P2 position. These data are in good agreement with previously published reports, in which some selected unnatural amino acids were used. For example, Tran et al. found that P1-position homophenylalanine in the fluorogenic substrate Gly-hPhe-AMC is more efficiently hydrolyzed by bovine cathepsin C than its one-methylene-group-shorter natural homolog, phenylalanine (Gly-Phe-AMC) (Tran et al. 2002). In the same work, the authors demonstrated that bovine cathepsin C barely recognizes Gly at the P2 position, but quite efficiently recognizes Ala, Abu, Nva and Nle. These data are also in good agreement with our library screening results. In another approach, Li et al. (2009) designed efficient rhodamine-based substrates for human cathepsin C with hPhe or Bip in the P1 position and Abu in the P2 position. The overall preference for quite broad substrate specificity in the P1 position of mammalian orthologs and rather narrow substrate specificity in the P2 position can be explained by the analysis of available crystal structures (Turk et al. 2001; Dahl et al. 2001; Molgaard et al. 2007). The S1 pocket is located on the surface of the enzyme and is exposed to the solvent. Its large size demonstrates that it preferentially accommodates very bulky and hydrophobic residues, such as these found in our studies. We assume that the large size of the residues results in more interactions with the surface of the enzyme and thus increases its affinity to the substrate. This hypothesis can by confirmed by the kinetic data we obtained for NH_2_-Abu-Nle(*O*-Bzl)-ACC and NH_2_-Gly-Phe-ACC, which differ very significantly in terms of the *k*_cat_/*K*_m_ value, but not as significantly in the *k*_cat_ value (Table 3). The determining factor here is the *K*_m_ value, which reflects, to a significant extent, the binding affinity of the substrate. Further analysis of the human cathepsin C crystal structure demonstrates that the S2 pocket is rather long and narrow. This explains why rather small and aliphatic amino acids, such as Ala, Abu, Met, Hse, Nle or Nva, are preferred in the P2 position. The structure of the optimal substrate NH_2_-Abu-Nle(*O*-Bzl)-ACC can also be used in further studies for the design of inhibitors or activity-based probes for cathepsin C. The observed substrate specificity also demonstrates that bovine cathepsin C, which is much more readily available and less expensive, can substitute for the human ortholog in routine studies on new chemical tools.

In the studies with malarial DPAP1, we provide new evidence that this enzyme quite significantly differs in substrate specificity from mammalian orthologs. In contrast to human and bovine cathepsin C, DPAP1 demonstrates very little preference in the P1 position toward bulky and hydrophobic amino acids of phenylalanine like structure and efficiently hydrolyzes amino acids with long aliphatic side chains with aromatic group at the end (Nle(*O*-Bzl), Glu(Bzl)) or with basic side chains, Arg and Lys. As a result of library screening studies, we have found that DPAP1 preferentially cleaves the unnatural cyclic amino acid Pip in the P2 position, which is barely tolerated by human orthologs. These results allowed us to design an optimal DPAP1 substrate, Pip-Lys-ACC, for which the *k*_cat_/*K*_m_ values were calculated. With *k*_cat_/*K*_m_ values equal to 6.59 × 10^5^ s^−1^ M^−1^ (DPAP1) and 0.96 × 10^5^ s^−1^ M^−1^ (cathepsin C), we have demonstrated that this substrate is quite selective toward the malarial ortholog. On the other hand, this might seem quite low given the huge difference in the initial screen. The reason is that DPAP1 is 30–100 times less efficient than cathepsin C using natural amino acid dipeptide substrates, as demonstrated by Wang et al. (2011). It is then apparent that the specificity has been shifted ~100-fold in favor of DPAP1.

The analyses presented here focused on the S1 and S2 pockets, but it is also likely that enhanced interactions with natural peptide and protein substrates utilize interactions at the C-terminal side (S′ side) of the scissile bond. Unfortunately, there is no currently available technology to probe the S′ site of aminopeptidases with synthetic substrates.

In conclusion, we have designed and tested for the first time a tailored fluorogenic substrate library containing natural and unnatural amino acids to define the specificity of the active site of human, bovine and malarial cathepsin C. Our results clearly demonstrate very significant differences in the preference for binding of both natural and unnatural amino acids to the S1 and S2 pockets between mammalian and malarial orthologs. The catalytic rates of hydrolysis for substrates with unnatural amino acids designed based on library screening were significantly improved, proving the utility of this approach. For example, for human cathepsin C, we have obtained a substrate that is more than 400 times better in terms of *k*_cat_/*K*_m_ than the commonly used commercial substrate. The observed significant differences in terms of the structure of the substrates between human and malarial orthologs can be used for the design of specific inhibitors or activity-based probes (ABPs). Finally, the methodology used here provides the proof of concept for the application of this library in screening other types of diaminopeptidases or for the direct comparison of enzyme orthologs from different organisms.

## Electronic supplementary material

Below is the link to the electronic supplementary material.

Supplementary material 1 (PDF 1278 kb)

Supplementary material 2 (PDF 164 kb)
