# Supplementary material for: Unnatural amino acids increase activity and specificity of synthetic substrates for human and malarial cathepsin C
Source: Amino Acids. 2014 Jan 1;46(4):931–43. doi: 10.1007/s00726-013-1654-2 (PMC3962583; doi:10.1007/s00726-013-1654-2)
Supplement: Supplementary file 1 — Supplementary material 1 (PDF 1278 kb) [file 726_2013_1654_MOESM1_ESM.pdf]

# Unnatural amino acids increase activity and specificity of synthetic substrates for human and malarial cathepsin C

Marcin Poreba[1], Marko Mihelic[2], Priscilla Krai[3], Jelena Rajkovic[2], Artur Krezel[4], Malgorzata Pawelczak[5], Michael Klemba[3], Dusan Turk[2], Boris Turk[2], Rafal Latajka[1], Marcin Drag[1,*]

[1]*Division of Bioorganic Chemistry, Faculty of Chemistry, Wroclaw University of Technology, Wybrzeze Wyspianskiego 27, 50-370 Wroclaw, Poland;* [2] *Department of Biochemistry and Molecular and Structural Biology, Jozef Stefan Institute, Ljubljana, Slovenia;* [3]*Department of Biochemistry, Virginia Tech, Blacksburg, VA, USA 24061;* [4]*Laboratory of Chemical Biology, Faculty of Biotechnology, University of Wrocław ul. Joliot-Curie 14a, 50-383 Wrocław, Poland;* [5]*Faculty of Chemistry, University of Opole, ul. Oleska 48, 45-052 Opole, Poland;*

# Supplemental data

**Table S1** Kinetic parameters ($K_m$, $k_{cat}$, $k_{cat}/K_m$) of two fluorogenic substrates determined in a three different pH conditions. Each experiment was repeated at least three times.

| | **pH = 5.0** | **pH = 5.5** | **pH = 6.0** |
|---|---|---|---|
| **$NH_2$-Abu-Nle(OBzl)-ACC** | | | |
| $K_m$, μM | 1.62 ± 0.09 | 1.88 ± 0.11 | 2.52 ± 0.21 |
| $k_{cat}$, $s^{-1}$ | 11.4 ± 0.32 | 17.8 ± 0.56 | 24.3 ± 1.20 |
| $k_{cat}/K_m$, $s^{-1}M^{-1}$ | 71.4 ± 3.56 | 94.5 ± 0.34 | 96.6 ± 1.23 |
| **Pip-Lys-ACC** | | | |
| $K_m$, μM | 87.1 ± 0.89 | 77.4 ± 5.35 | 87.6 ± 2.85 |
| $k_{cat}$, $s^{-1}$ | 5.65 ± 0.21 | 7.45 ± 0.41 | 9.27 ± 0.22 |
| $k_{cat}/K_m$, $s^{-1}M^{-1}$ | 0.62 ± 0.02 | 0.96 ± 0.07 | 1.03 ± 0.11 |

**Table S2** Kinetic parameters ($K_m$, $k_{cat}$, $k_{cat}/K_m$) of two fluorogenic substrates determined in a four different DTT concentrations. Each experiment was repeated at least three times.

| | **[DDT] = 0 mM** | **[DDT] = 1 mM** | **[DDT] = 5 mM** | **[DDT] = 10 mM** |
|---|---|---|---|---|
| **$NH_2$-Abu-Nle(OBzl)-ACC** | | | | |
| $K_m$, μM | 2.47 ± 0.35 | 2.47 ± 0.18 | 1.88 ± 0.11 | 1.94 ± 0.12 |
| $k_{cat}$, $s^{-1}$ | 0.65 ± 0.12 | 15.5 ± 0.25 | 17.8 ± 0.56 | 18.3 ± 0.22 |
| $k_{cat}/K_m$, $s^{-1}M^{-1}$ | 26.3 ± 1.95 | 62.7 ± 3.41 | 94.5 ± 0.34 | 94.4 ± 0.69 |
| **Pip-Lys-ACC** | | | | |
| $K_m$, μM | 64.5 ± 0.35 | 92.9 ± 2.56 | 77.4 ± 5.35 | 95.8 ± 5.65 |
| $k_{cat}$, $s^{-1}$ | 0.82 ± 0.09 | 9.27 ± 0.52 | 7.45 ± 0.41 | 11.7 ± 0.12 |
| $k_{cat}/K_m$, $s^{-1}M^{-1}$ | 0.13 ± 0.02 | 0.99 ± 0.10 | 0.96 ± 0.07 | 1.13 ± 0.01 |

**Table S3** Kinetic parameters ($K_m$, $k_{cat}$, $k_{cat}/K_m$) of two fluorogenic substrates determined in a five different NaCl concentrations. Each experiment was repeated at least three times.

| | **[NaCl] = 0 mM** | **[NaCl] = 5 mM** | **[NaCl] = 30 mM** | **[NaCl] = 100 mM** | **[NaCl] = 500 mM** | **[NaCl] = 1000 mM** |
|---|---|---|---|---|---|---|
| **$NH_2$-Abu-Nle(OBzl)-ACC** | | | | | | |
| $K_m$, μM | > 10 | 3.87 ± 0.23 | 1.79 ± 0.08 | 1.88 ± 0.11 | 2.63 ± 0.21 | 3.09 ± 0.15 |
| $k_{cat}$, $s^{-1}$ | < 5 | 19.3 ± 1.10 | 17.5 ± 0.33 | 17.8 ± 0.56 | 22.3 ± 1.55 | 21.8 ± 1.25 |
| $k_{cat}/K_m$, $s^{-1}M^{-1}$ | 4.51 ± 0.56 | 49.7 ± 3.92 | 97.8 ± 6.32 | 94.5 ± 0.34 | 84.8 ± 5.64 | 70.5 ± 6.35 |
| **Pip-Lys-ACC** | | | | | | |
| $K_m$, μM | 102.8 ± 12.3 | 101.1 ± 5.21 | 83.1 ± 5.56 | 77.4 ± 5.35 | 114.7 ± 9.63 | 127.8 ± 5.64 |
| $k_{cat}$, $s^{-1}$ | 1.11 ± 0.08 | 5.03 ± 0.26 | 7.60 ± 0.68 | 7.45 ± 0.41 | 6.27 ± 0.88 | 5.25 ± 0.22 |
| $k_{cat}/K_m$, $s^{-1}M^{-1}$ | 0.11 ± 0.02 | 0.49 ± 0.06 | 1.09 ± 0.12 | 0.96 ± 0.07 | 0.55 ± 0.05 | 0.39 ± 0.05 |

**Table S4** Kinetic parameters ($K_m$, $k_{cat}$, $k_{cat}/K_m$) of two fluorogenic substrates determined in two different buffers.

| | **Human cathepsin C buffer** | ***Plasmodium falciparum* DPAP1 buffer** |
|---|---|---|
| **$NH_2$-Abu-Nle(OBzl)-ACC** | | |
| $K_m$, μM | 1.88 ± 0.11 | 1.86 ± 0.58 |
| $k_{cat}$, $s^{-1}$ | 17.8 ± 0.56 | 22.1 ± 1.59 |
| $k_{cat}/K_m$, $s^{-1}M^{-1}$ | 94.5 ± 0.34 | 108.5 ± 5.68 |
| **Pip-Lys-ACC** | | |
| $K_m$, μM | 77.4 ± 5.35 | 69.4 ± 5.64 |
| $k_{cat}$, $s^{-1}$ | 7.45 ± 0.41 | 8.47 ± 1.10 |
| $k_{cat}/K_m$, $s^{-1}M^{-1}$ | 0.96 ± 0.07 | 1.08 ± 0.08 |

P1 LIBRARY

| Entry | Structure | Name | M.W. | P1 side chain |
|---|---|---|---|---|
| **1.** | 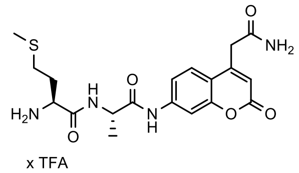 | **$NH_2$-*L*-Met-*L*-Ala-ACC** | **534.51** | *L*-alanine |
| **2.** | 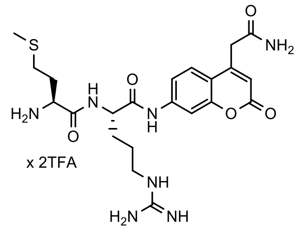 | **$NH_2$-*L*-Met-*L*-Arg-ACC** | **733.64** | *L*-arginine |
| **3.** | 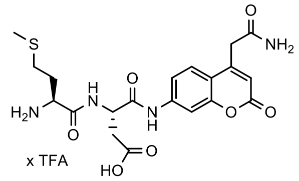 | **$NH_2$-*L*-Met-*L*-Asp-ACC** | **578.52** | *L*-aspartic acid |
| **4.** | 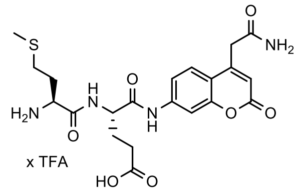 | **$NH_2$-*L*-Met-*L*-Glu-ACC** | **592.54** | *L*- glutamic acid |
| **5.** | 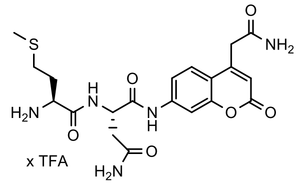 | **$NH_2$-*L*-Met-*L*-Asn-ACC** | **577.53** | *L*-asparagine |
| **6.** | 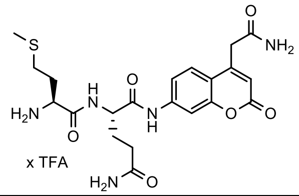 | **$NH_2$-*L*-Met-*L*-Gln-ACC** | **591.56** | *L*-glutamine |
| **7.** | 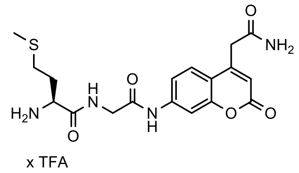 | **$NH_2$-*L*-Met-Gly-ACC** | **520.48** | glycine |
| **8.** | 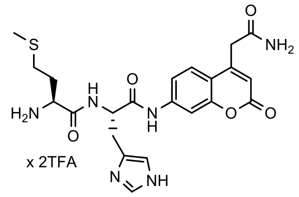 | **$NH_2$-*L*-Met-*L*-His-ACC** | **714.59** | *L*-histidine |
| **9.** | 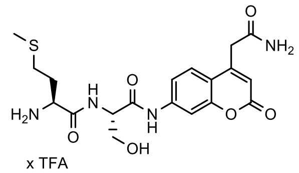 | **$NH_2$-*L*-Met-*L*-Ser-ACC** | **550.51** | *L*-serine |

| | | | | |
|---|---|---|---|---|
| **10.** | 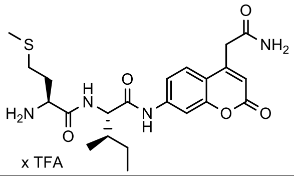 | **NH$_2$-*L*-Met-*L*-Ile-ACC** | **576.59** | *L*-isoleucine |
| **11.** | 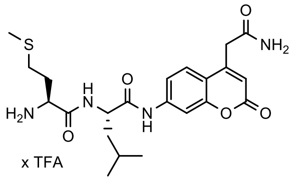 | **NH$_2$-*L*-Met-*L*-Leu-ACC** | **576.59** | *L*-leucine |
| **12.** | 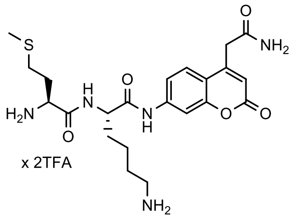 | **NH$_2$-*L*-Met-*L*-Lys-ACC** | **705.62** | *L*-lysine |
| **13.** | 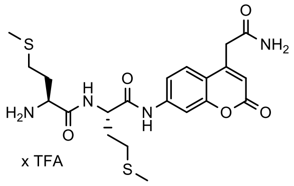 | **NH$_2$-*L*-Met-*L*-Met-ACC** | **594.62** | *L*-methionine |
| **14.** | 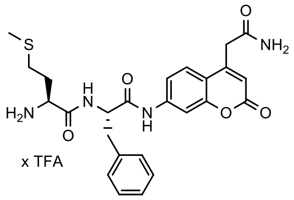 | **NH$_2$-*L*-Met-*L*-Phe-ACC** | **610.60** | *L*-phenylalanine |
| **15.** | 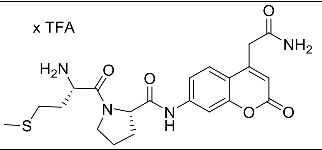 | **NH$_2$-*L*-Met-*L*-Pro-ACC** | **560.54** | *L*-proline |
| **16.** | 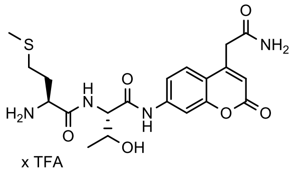 | **NH$_2$-*L*-Met-*L*-Thr-ACC** | **564.53** | *L*-threonine |
| **17.** | 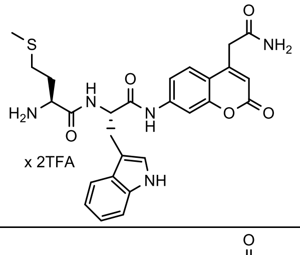 | **NH$_2$-*L*-Met-*L*-Trp-ACC** | **763.66** | *L*-tryptophan |
| **18.** | 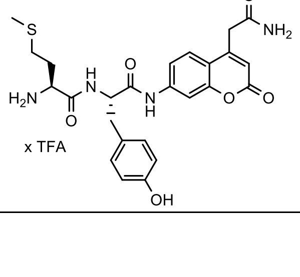 | **NH$_2$-*L*-Met-*L*-Tyr-ACC** | **626.60** | *L*-tyrosine |

| | | | | |
|---|---|---|---|---|
| **19.** | 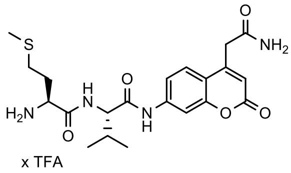 | **NH$_2$-*L*-Met-*L*-Val-ACC** | **562.56** | *L*-valine |
| **20.** | 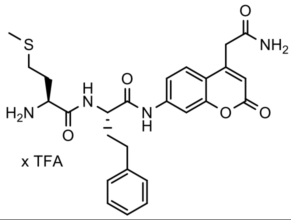 | **NH$_2$-*L*-Met-*L*-hPhe-ACC** | **624.63** | *L*-homophenylalanine |
| **21.** | 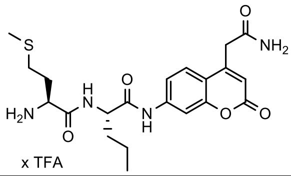 | **NH$_2$-*L*-Met-*L*-Nva-ACC** | **562.56** | *L*-norvaline |
| **22.** | 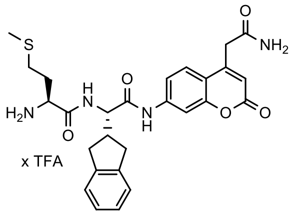 | **NH$_2$-*L*-Met-*L*-Igl-ACC** | **636.64** | *L*-indanylglycine |
| **23.** | 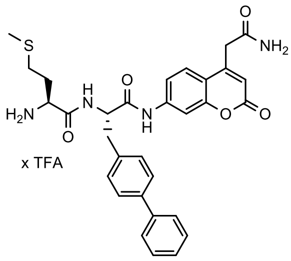 | **NH$_2$-*L*-Met-*L*-Bip-ACC** | **686.70** | *L*-biphenylalanine |
| **24.** | 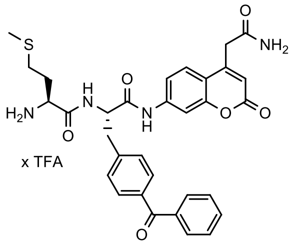 | **NH$_2$-*L*-Met-*L*-Bpa-ACC** | **714.71** | *L*-(4-benzoyl)-phenylalanine |
| **25.** | 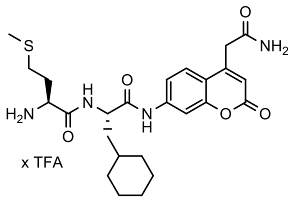 | **NH$_2$-*L*-Met-*L*-Cha-ACC** | **616.65** | *L*-cyclohexylalanine |
| **26.** | 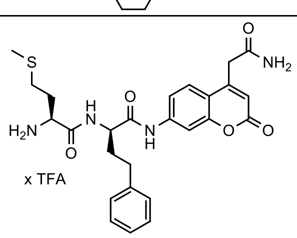 | **NH$_2$-*L*-Met-*D*-hPhe-ACC** | **624.63** | *D*-homophenylalanine |

| | | | | |
|---|---|---|---|---|
| **27.** | 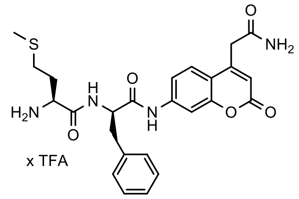 | **NH$_2$-*L*-Met-*D*-Phe-ACC** | **610.6** | *D*-phenylalanine |
| **28.** | 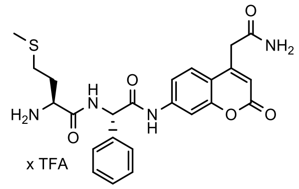 | **NH$_2$-*L*-Met-*L*-Phg-ACC** | **596.58** | *L*-phenylglycine |
| **29.** | 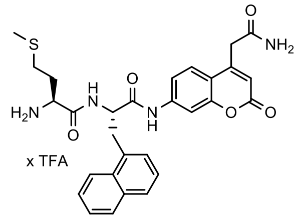 | **NH$_2$-*L*-Met-*L*-1-Nal-ACC** | **660.66** | *L*-(1-naphthyl)-alanine |
| **30.** | 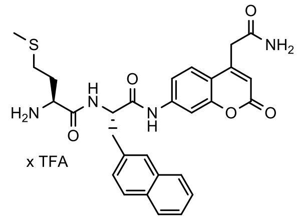 | **NH$_2$-*L*-Met-*L*-2-Nal-ACC** | **660.66** | *L*-(2-naphthyl)-alanine |
| **31.** | 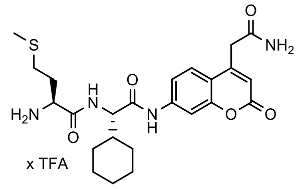 | **NH$_2$-*L*-Met-*L*-Chg-ACC** | **602.62** | *L*-cyclohexylglycine |
| **32.** | 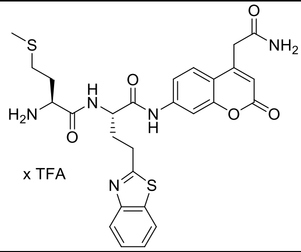 | **NH$_2$-*L*-Met-*L*-Abu(Bht)-ACC** | **681.70** | *L*-(4-benzothiazol-2-yl)homoalanine |
| **33.** | 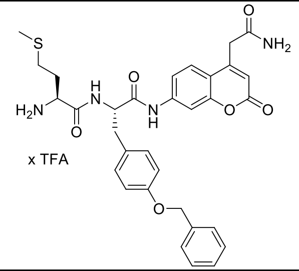 | **NH$_2$-*L*-Met-*L*-Tyr(Bzl)-ACC** | **716.72** | *L*-tyrosine *O*-benzyl ester |
| **34.** | 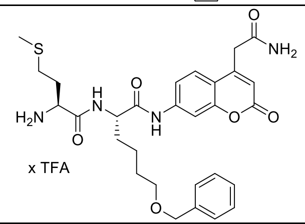 | **NH$_2$-*L*-Met-*L*-Nle(6-*O*-Bzl)-ACC** | **682.71** | *L*-(6-benzyloxy)-norleucine |

| | | | | |
|---|---|---|---|---|
| **35.** | 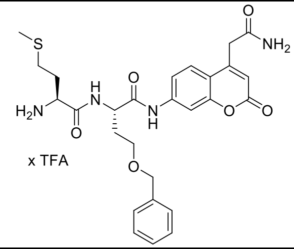 | **NH$_2$-*L*-Met-*L*-Hse(Bzl)-ACC** | **654.65** | *L*-homoserine-*O*-benzyl ester |
| **36.** | 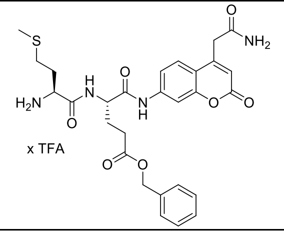 | **NH$_2$-*L*-Met-*L*-Glu(Bzl)-ACC** | **682.66** | *L*-glutamic acid *O*-benzyl ester |

P2 LIBRARY

| Entry | Structure | Name | M.W. | P2 side chain |
|---|---|---|---|---|
| **1.** | 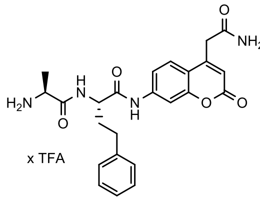 | **NH$_2$-*L*-Ala-*L*-hPhe-ACC** | **564.51** | *L*-alanine |
| **2.** | 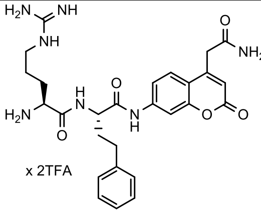 | **NH$_2$-*L*-Arg-*L*-hPhe-ACC** | **763.64** | *L*-arginine |
| **3.** | 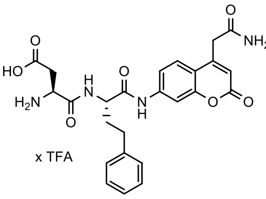 | **NH$_2$-*L*-Asp-*L*-hPhe-ACC** | **608.52** | *L*-aspartic acid |
| **4.** | 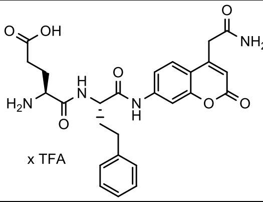 | **NH$_2$-*L*-Glu-*L*-hPhe-ACC** | **622.55** | *L*-glutamic acid |
| **5.** | 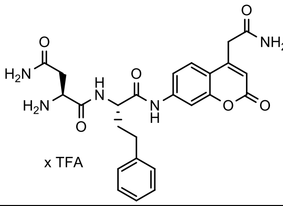 | **NH$_2$-*L*-Asn-*L*-hPhe-ACC** | **607.54** | *L*-asparagine |

| | | | | |
|---|---|---|---|---|
| **6.** | 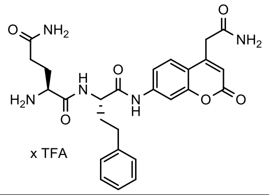 | **NH$_2$-*L*-Gln-*L*-hPhe-ACC** | **621.56** | *L*-glutamine |
| **7.** | 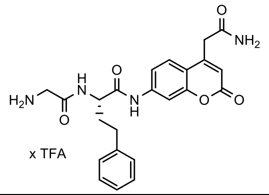 | **NH$_2$-Gly-*L*-hPhe-ACC** | **550.48** | glycine |
| **8.** | 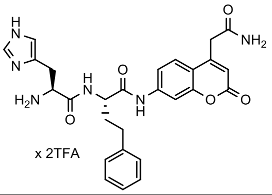 | **NH$_2$-*L*-His-*L*-hPhe-ACC** | **744.60** | *L*-histidine |
| **9.** | 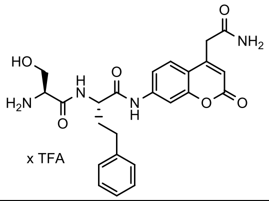 | **NH$_2$-*L*-Ser-*L*-hPhe-ACC** | **580.51** | *L*-serine |
| **10.** | 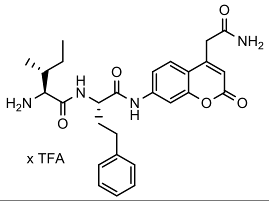 | **NH$_2$-*L*-Ile-*L*-hPhe-ACC** | **606.59** | *L*-isoleucine |
| **11.** | 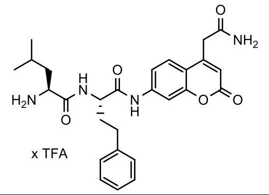 | **NH$_2$-*L*-Leu-*L*-hPhe-ACC** | **606.59** | *L*-leucine |
| **12.** | 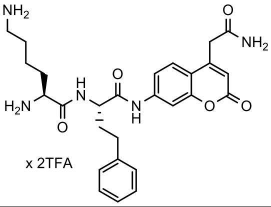 | **NH$_2$-*L*-Lys-*L*-hPhe-ACC** | **735.63** | *L*-lysine |
| **13.** | 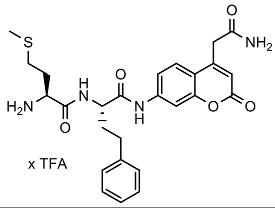 | **NH$_2$-*L*-Met-*L*-hPhe-ACC** | **624.63** | *L*-methionine |
| **14.** | 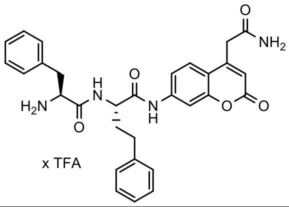 | **NH$_2$-*L*-Phe-*L*-hPhe-ACC** | **640.61** | *L*-phenylalanine |

| | | | | |
|---|---|---|---|---|
| **15.** | 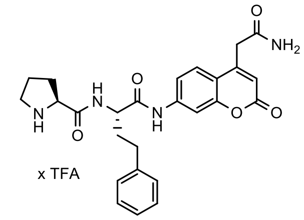 | ***L*-Pro-*L*-hPhe-ACC** | **590.55** | *L*-proline |
| **16.** | 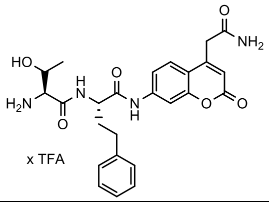 | **$NH_2$-*L*-Thr-*L*-hPhe-ACC** | **594.54** | *L*-threonine |
| **17.** | 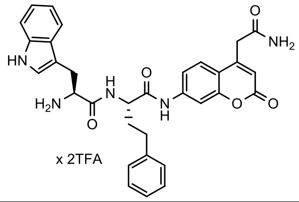 | **$NH_2$-*L*-Trp-*L*-hPhe-ACC** | **793.67** | *L*-tryptophan |
| **18.** | 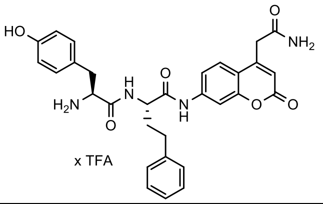 | **$NH_2$-*L*-Tyr-*L*-hPhe-ACC** | **656.61** | *L*-tyrosine |
| **19.** | 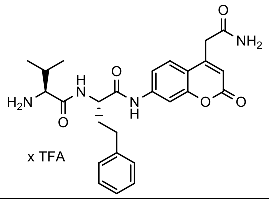 | **$NH_2$-*L*-Val-*L*-hPhe-ACC** | **592.56** | *L*-valine |
| **20.** | 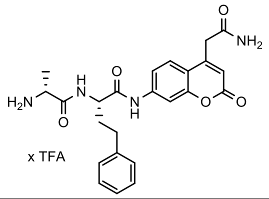 | **$NH_2$-*D*-Ala-*L*-hPhe-ACC** | **564.51** | *D*-alanine |
| **21.** | 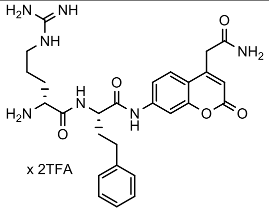 | **$NH_2$-*D*-Arg-*L*-hPhe-ACC** | **763.64** | *D*-arginine |
| **22.** | 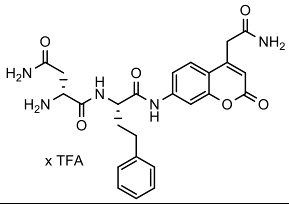 | **$NH_2$-*D*-Asn-*L*-hPhe-ACC** | **607.54** | *D*-asparagine |
| **23.** | 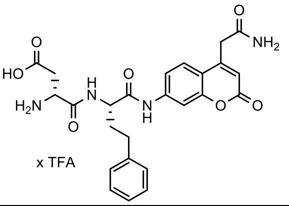 | **$NH_2$-*D*-Asp-*L*-hPhe-ACC** | **608.52** | *D*-aspartic acid |

| | | | | |
|---|---|---|---|---|
| **24.** | 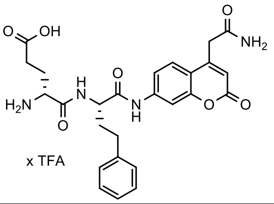 | **NH$_2$-*D*-Glu-*L*-hPhe-ACC** | **622.55** | *D*-glutamic acid |
| **25.** | 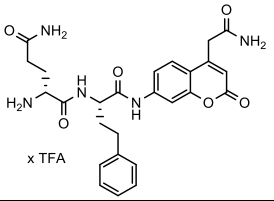 | **NH$_2$-*D*-Gln-*L*-hPhe-ACC** | **621.56** | *D*-glutamine |
| **26.** | 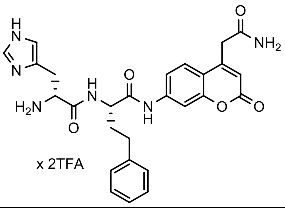 | **NH$_2$-*D*-His-*L*-hPhe-ACC** | **744.60** | *D*-histidine |
| **27.** | 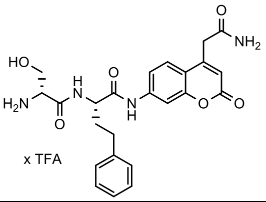 | **NH$_2$-*D*-Ser-*L*-hPhe-ACC** | **580.51** | *D*-serine |
| **28.** | 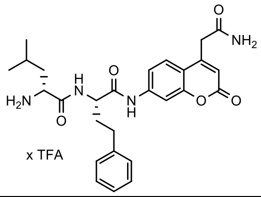 | **NH$_2$-*D*-Leu-*L*-hPhe-ACC** | **606.59** | *D*-leucine |
| **29.** | 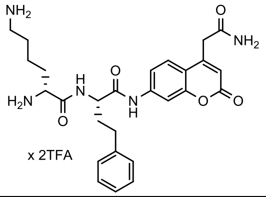 | **NH$_2$-*D*-Lys-*L*-hPhe-ACC** | **735.63** | *D*-lysine |
| **30.** | 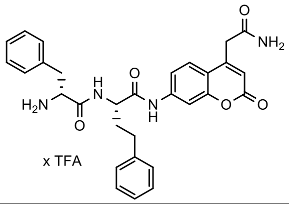 | **NH$_2$-*D*-Phe-*L*-hPhe-ACC** | **640.61** | *D*-phenylalanine |
| **31.** | 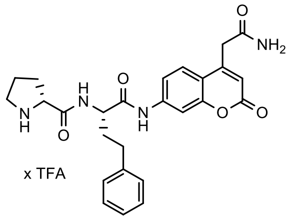 | ***D*-Pro-*L*-hPhe-ACC** | **590.55** | *D*-proline |
| **32.** | 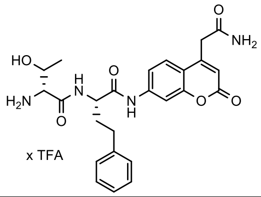 | **NH$_2$-*D*-Thr-*L*-hPhe-ACC** | **594.54** | *D*-threonine |

| | | | | |
|---|---|---|---|---|
| **33.** | 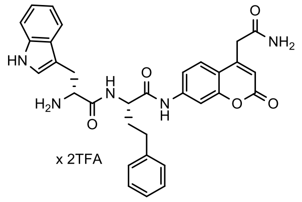 | **NH$_2$-*D*-Trp-*L*-hPhe-ACC** | **793.67** | *D*-tryptophan |
| **34.** | 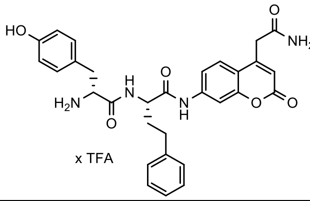 | **NH$_2$-*D*-Tyr-*L*-hPhe-ACC** | **656.61** | *D*-tyrosine |
| **35.** | 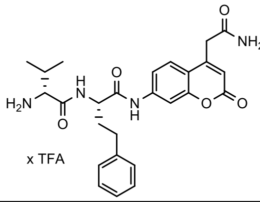 | **NH$_2$-*D*-Val-*L*-hPhe-ACC** | **592.56** | *D*-valine |
| **36.** | 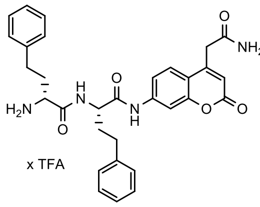 | **NH$_2$-*D*-hPhe-*L*-hPhe-ACC** | **654.63** | *D*-homophenylalanine |
| **37.** | 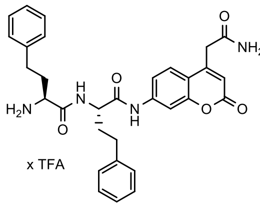 | **NH$_2$-*L*-hPhe-*L*-hPhe-ACC** | **654.63** | *L*-homophenylalanine |
| **38.** | 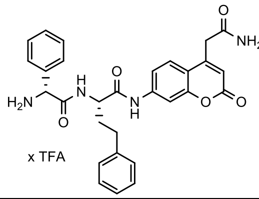 | **NH$_2$-*D*-Phg-*L*-hPhe-ACC** | **626.58** | *D*-phenylglycine |
| **39.** | 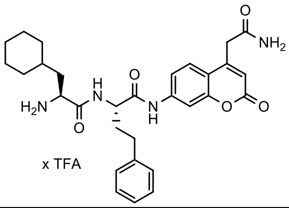 | **NH$_2$-*L*-Cha-*L*-hPhe-ACC** | **646.65** | *L*-cyclohexylalanine |
| **40.** | 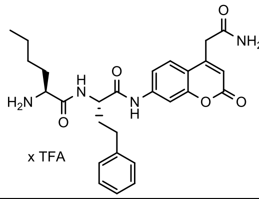 | **NH$_2$-*L*-Nva-*L*-hPhe-ACC** | **606.59** | *L*-norleucine |
| **41.** | 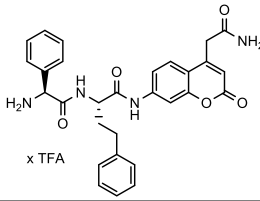 | **NH$_2$-*L*-Phg-*L*-hPhe-ACC** | **626.58** | *L*-phenylglycine |

| | | | | |
|---|---|---|---|---|
| **42.** | 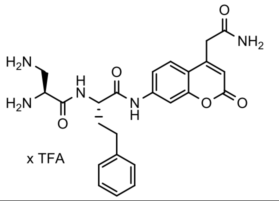 | **NH$_2$-*L*-Dap-*L*-hPhe-ACC** | **693.55** | *L*-aminoalanine |
| **43.** | 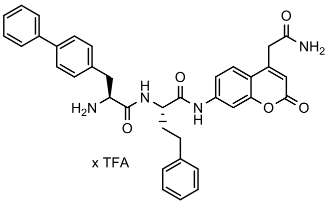 | **NH$_2$-*L*-Bip-*L*-hPhe-ACC** | **716.70** | *L*-biphenylalanine |
| **44.** | 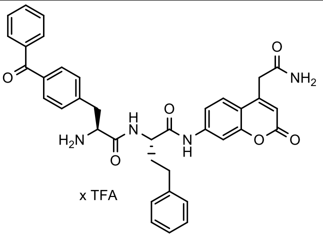 | **NH$_2$-*L*-Bpa-*L*-hPhe-ACC** | **744.71** | *L*-(4-benzoyl)-phenylalanine |
| **45.** | 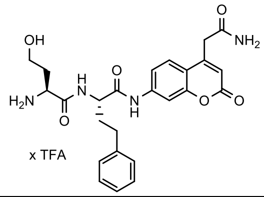 | **NH$_2$-*L*-Hse-*L*-hPhe-ACC** | **594.54** | *L*-homoserine |
| **46.** | 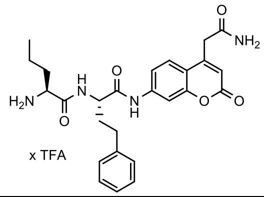 | **NH$_2$-*L*-Nva-*L*-hPhe-ACC** | **592.56** | *L*-norvaline |
| **47.** | 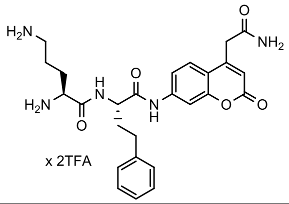 | **NH$_2$-*L*-Orn-*L*-hPhe-ACC** | **721.60** | *L*-ornithine |
| **48.** | 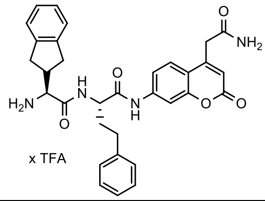 | **NH$_2$-*L*-Igl-*L*-hPhe-ACC** | **666.64** | *L*-indanylglycine |
| **49.** | 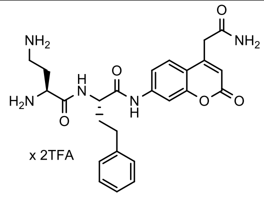 | **NH$_2$-*L*-Dab-*L*-hPhe-ACC** | **707.57** | *L*-aminohomoalanine |

| | | | | |
|---|---|---|---|---|
| **50.** | 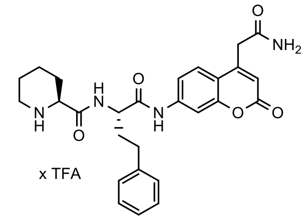 | ***L*-Pip-*L*-hPhe-ACC** | **640.57** | *L*-piperidine |
| **51.** | 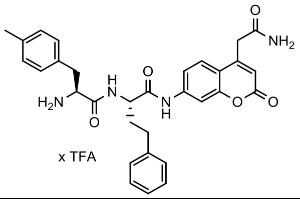 | **$NH_2$-*L*-4-$CH_3$-Phe-*L*-hPhe-ACC** | **654.63** | *L*-(4-methyl)-phenylalanine |
| **52.** | 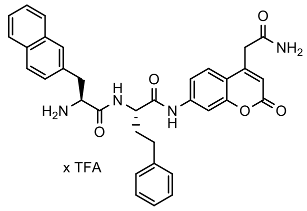 | **$NH_2$-*L*-2-Nal-*L*-hPhe-ACC** | **690.67** | *L*-(2-naphthyl)-alanine |
| **53.** | 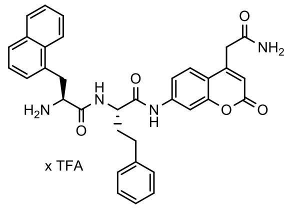 | **$NH_2$-*L*-1-Nal-*L*-hPhe-ACC** | **690.67** | *L*-(1-naphthyl)-alanine |
| **54.** | 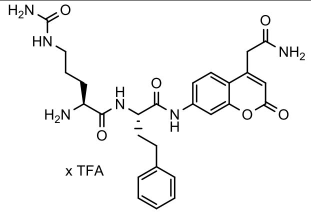 | **$NH_2$-*L*-Cit-*L*-hPhe-ACC** | **650.60** | *L*-citrulline |
| **55.** | 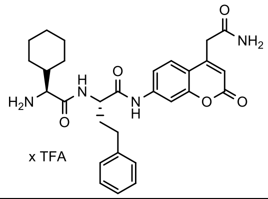 | **$NH_2$-*L*-Chg-*L*-hPhe-ACC** | **632.63** | *L*-cyclohexylglycine |
| **56.** | 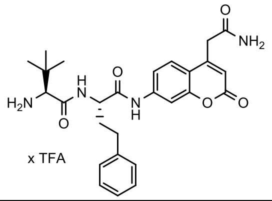 | **$NH_2$-*L*-Tle-*L*-hPhe-ACC** | **606.59** | *L*-*tert*-leucine |
| **57.** | 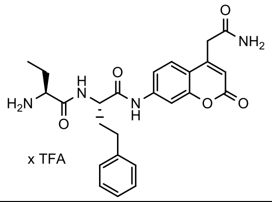 | **$NH_2$-*L*-Abu-*L*-hPhe-ACC** | **578.54** | *L*-homoalanine |

Additional fluorogenic substrates

| Entry | Structure | Name | M.W. | P2/P1 side chains |
|---|---|---|---|---|
| **1.** | 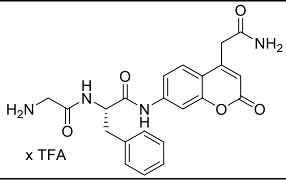 | **$NH_2$-Gly-*L*-Phe-ACC** | **536.46** | P1: *L*-phenylalanine<br>P2: glycine |
| **2.** | 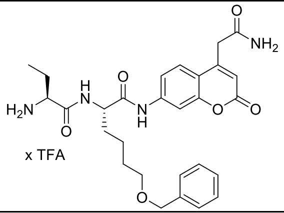 | **$NH_2$-*L*-Abu-*L*-Nle-(*O*-Bzl)-ACC** | **636.62** | P1: *L*-(6-benzyloxy)-norleucine<br>P2: *L*-homoalanine |
| **3.** | 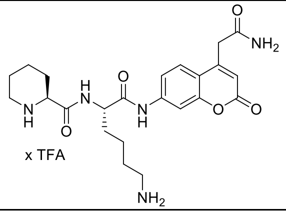 | ***L*-Pip-*L*-Lys-ACC** | **571.55** | P1: *L*-lysine<br>P2: *L*-piperidine |
