# Supplementary material for: Unnatural amino acids increase activity and specificity of synthetic substrates for human and malarial cathepsin C
Source: Amino Acids. 2014 Jan 1;46(4):931–43. doi: 10.1007/s00726-013-1654-2 (PMC3962583; doi:10.1007/s00726-013-1654-2)
Supplement: Supplementary file 2 — Supplementary material 2 (PDF 164 kb) [file 726_2013_1654_MOESM2_ESM.pdf]

Activation of Procathepsin C

Procathepsin C
- A280 = 2.477
- ε = 99000

→ 25 μM

- 4 ml of Procathepsin C (in 30 mM Tris, 0.4 M NaCl, pH 7.5) was mixed with 1 M NaOAC, pH 5.0 buffer
- 800 μl of cathepsin L was added (final molar ration CatC/CatL = 10/1)
- 1 M DTT was added to 5 mM final concentration
- mixture was incubated at 37°C
- in 15 min time intervals, activity agains Z-Gly-Phe-pNA was measured
- when max. activity was reached, the mature cathepsin C was separated from cathepsin L on Superdex S200 HR column
- protein peak with activity against Z-Gly-Phe-pNA was collected

Active site titration of Cathepsin L

Cathepsin L
- A280 = 0.254
- ε = 47100

→ 5.392 μM

Reaction buffer:
- 0.1 M NaOAc
- 1 mM EDTA
- 5 mM DTT
- pH 5.5

All dilutions and measurements were done in "Reaction buffer"

E-64:
- stock solution: 50 mM in DMSO

Substrate:
- Z-Phe-Arg-pNA
- 0.1 M in DMSO

Reaction setup:
- 53.93 nM solution of cathepsin L (final concentration) was mixed with increasing concentrations of E-64 in total volume of 500 μl. After 15 minutes of incubation at room temperature, 500 μl of 200 μM solution of substrate was added and increase of absorbance at 410 nm was measured for 1 minute (in 1 sec. intervals).

Results:

- Cathepsin L is 65 % active
- Active concentration of Cathepsin L is **3.5048 μM**

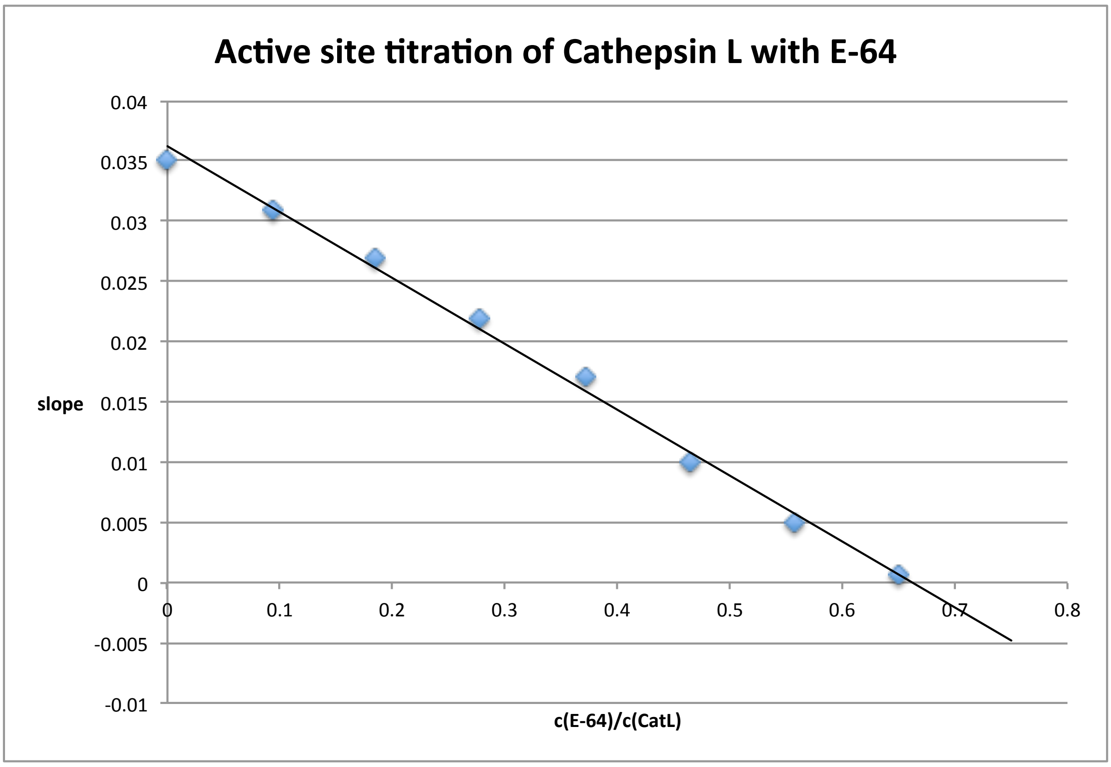

Active site titration of Cystatin C

Cystatin C

- A280 = 1.720
- ε = 11710

→ 146.88 μM

Cathepsin L

→ 3.5048 μM

Reaction buffer:

- 0.1 M NaOAc
- 1 mM EDTA
- 5 mM DTT
- pH 5.5

All dilutions and measurements were done in "Reaction buffer"

Substrate:

- Z-Phe-Arg-pNA
- 0.1 M in DMSO

Reaction setup:

- 17.7969 nM solution of cathepsin L (final concentration) was mixed with increasing concentrations of Cystatin C to a final volume 950 μl. After 30 min of incubation at room temperature, 50 μl of 2 mM solution of substrate was added and increase of absorbance at 410 nm was measured for 1 minute (in 1 sec. intervals).
- two independent parallel experiments were performed

Results:

- Cystatin C is approx. 61 % active
- Active concentration of Cystatin C is **89.5968 μM**

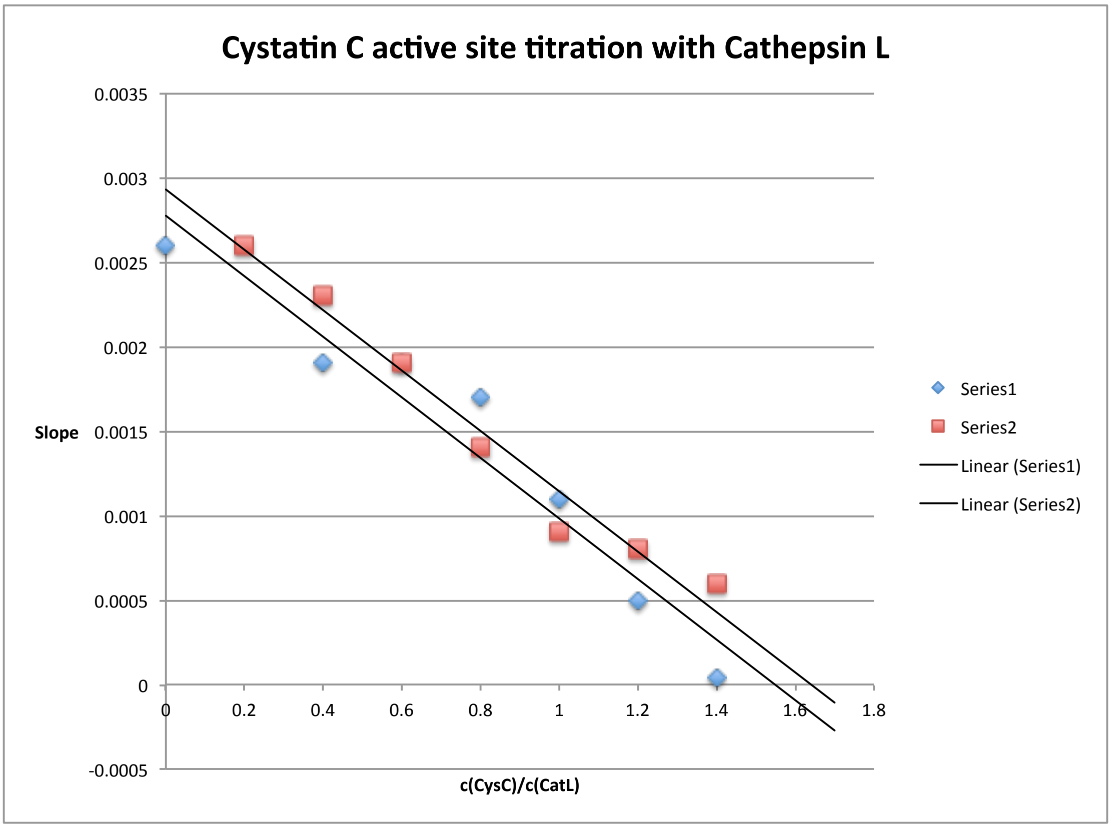

Active site titration of Cathepsin C with Cystatin C

Cathepsin C

- A280 = 0.31082
- ε = 84200

→ 3.691 μM

Reaction buffer:

- 0.1 M NaOAc
- 100 mM NaCl
- 1 mM EDTA
- 5 mM DTT
- pH 5.5

All dilutions and measurements were done in "Reaction buffer"

Cystatin C:

- 89.5968 μM

Substrate:

- Z-Gly-Phe-pNA
- 0.1 M in DMSO

Reaction setup:

- 369.1 nM solution of cathepsin C (final concentration) was mixed with increasing concentrations of Cystatin C in total volume of 950 μl. After 30 minutes of incubation at room temperature, 50 μl of 2 mM solution of substrate was added and increase of absorbance at 410 nm was measured for 3 minute (in 1 sec. intervals).

Results:

- Cathepsin C is 65 % active

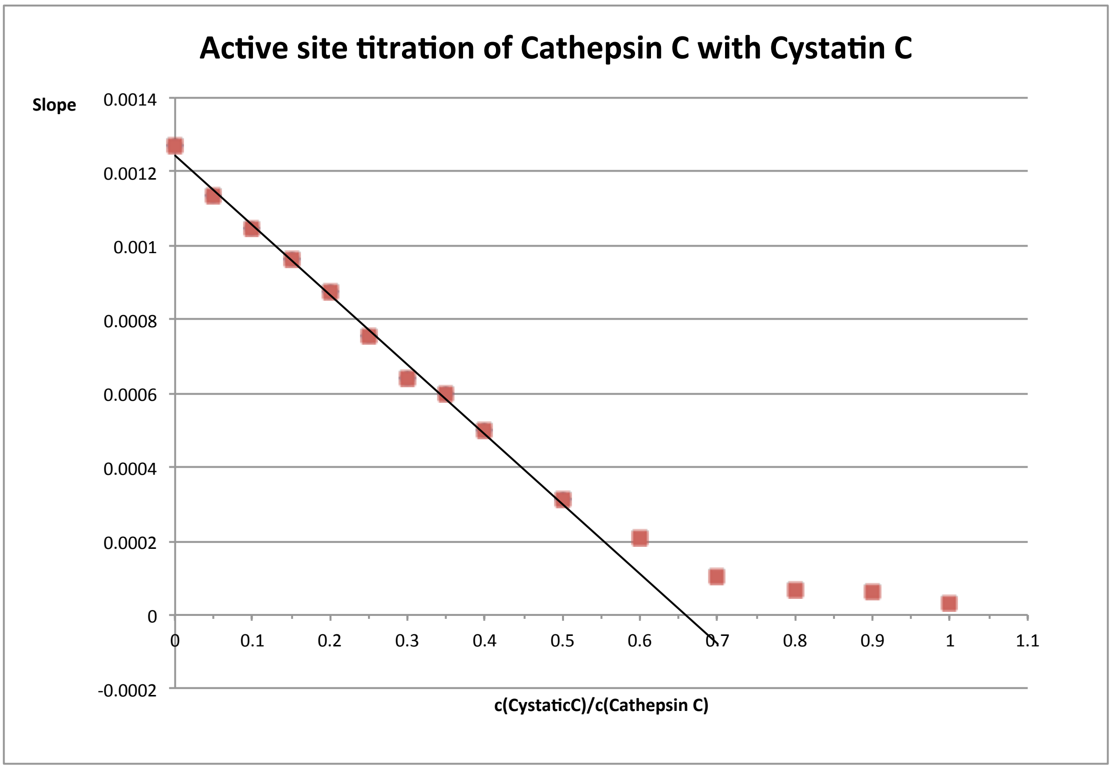

Active site titration of Cathepsin C with Cystatin C
Slope
0.0014
0.0012
0.001
0.0008
0.0006
0.0004
0.0002
0
-0.0002
0
0.1
0.2
0.3
0.4
0.5
0.6
0.7
0.8
0.9
1
1.1
c(CystaticC)/c(Cathepsin C)
